# The Analysis of a Causal Relationship between Career Maturity and Academic Achievement on Korean Adolescents Using Autoregressive Cross-Lagged Modeling

**DOI:** 10.3390/ijerph19095572

**Published:** 2022-05-04

**Authors:** Sung-Man Bae

**Affiliations:** Department of Psychology and Psychotherapy, College of Health Science, Dankook University, 119 Dandae-ro, Dongnam-gu, Cheonan-si 31116, Korea; spirit73@hanmail.net; Tel.: +82-550-8142

**Keywords:** career maturity, academic achievement, autoregressive effect, cross-lagged effect, bidirectional effect

## Abstract

Academic achievement and career maturity are closely related, but an understanding of the causal direction is lacking. The purpose of this study was to analyze the causal relationship between career maturity and academic achievement using autoregressive cross-lagged modeling. This study analyzed the data of 888 adolescents (mean age = 15.90) from the Youth Panel Survey. Autoregressive modeling indicates that academic achievement and career maturity remained stable over time. Higher academic achievement at a previous time point was associated with higher academic achievement at the next time point and similarly for career maturity. Moreover, as a result of cross-lagged effects, academic achievement at one time had a positive effect on career maturity at the next time point, while career maturity at one time had a positive impact on academic achievement at the next time point. In other words, there was a bidirectional effect between academic achievement and career maturity. This study implies that researchers and educators should consider career maturity as well as academic achievement for career guidance.

## 1. Introduction

Academic achievement is a major academic concern in adolescence and is closely associated with factors related to mental health status, such as perceived stress, depression, and anxiety. Based on Super’s career development theory and integrated contextual model (ICM), career maturity may promote school activation and participation, such as learning activities. A study of 174 college students has verified that career maturity positively predicts academic achievement [1]. A longitudinal study of Korean adolescents has identified that students’ perceptions of career maturity significantly predict academic achievement [2]. In addition, Oliveira et al. [3] verified the impact of career preparedness (expectations of the outcomes of career explorations and career planning) during middle-school years on academic achievement, using longitudinal trend articulations.

Previous studies have shown inconsistent results: Some studies indicate that career maturity affects academic achievement (e.g., [4]), while other studies argue that academic achievement is a key predictor of career maturity (e.g., [5]). Most studies involved a cross-sectional design, which is related to these discrepancies. Thus, an analysis adopting a longitudinal design such as an autoregressive cross-lagged model is needed to clarify the causal relationship between the two variables.

Many past studies were conducted for students in late childhood and early adolescence [6]. According to career development theory, the elementary and middle school years are usually a time of career growth. It is unusual, however, for students to have a high score on a career maturity scale during this period of time [7]. Therefore, longitudinal studies of high school students are needed to clarify the causal association between career maturity and academic achievement.

The aim of this study is to analyze the causal association between career maturity and academic achievement using a cross-lagged autoregressive model. The research questions of this study are as follows: First, are the autoregressive effects of academic achievement and career maturity stable over time? Second, do academic achievement and career maturity show cross-lagged effects over time? The main hypotheses are as follows: First, academic achievement will have a positive effect on career maturity over time. Second, career maturity will have a positive effect on academic achievement over time. Third, academic achievement and career maturity will have bidirectional influences over time.

## 2. Theoretical Framework

According to career development theory [8], career development is a continuous, lifelong process that is composed of five stages (growth: birth to 14 years; exploration: 14–25 years; establishment: 25–45 years; maintenance: 45–65 years; disengagement: 65 years and beyond), with career maturity as the core component of the process [9]. Career maturity includes an understanding of one’s own aptitudes and interests, and awareness of preferred career or occupation [10]. Particularly, students who have a high awareness of career options are likely to prepare for a specific career, be active in their school life [11], and have high academic performance [3]. The integrated contextual model (ICM) of career development [11] suggests that career development skills such as career exploration and planning may enhance school engagement and academic achievement [6,12].

There is an alternative model suggesting that career maturity is a result of high academic achievement [13]. Academic achievement may play an important role in choosing a student’s career [14]. Some studies have found significant differences in career maturity levels between groups with different levels of academic achievement [14]. In particular, Bae [5] examined the long-term relationship between academic achievement and career maturity using multivariate latent growth modeling while adjusting for gender and house income, finding that academic achievement was a key predictor of career maturity.

Students with higher academic achievement tend to explore their careers concretely and actively, while adolescents with lower academic achievement have low self-efficacy and are less active in career exploration [5]. In particular, Korean students are committed to achieving high scores on college entrance exams [13], but they face a lack of opportunities to explore and prepare for their future careers. In addition, because Korean society is an education-oriented society, students’ academic achievement has a great impact on their career choice.

## 3. Method

### 3.1. Design

A quantitative longitudinal analysis was employed to verify the causal relationship between variables. This study used the Youth Panel Survey data of the Korea Employment Information Service (KEIS). Based on the 2005 National Population Census, 3750 geographic areas and 9000 households were extracted using stratified random sampling. The survey was conducted by a computer-assisted personal interview (CAPI) method in which 87 trained interviewers visited the survey households directly. For participants who rejected the survey method or were difficult to contact, an online survey method using a CAPI questionnaire was used.

### 3.2. Participants

Of the total respondents, 95.9% of respondents used CAPI, and 4.1% used the online survey. In the first year, 888 adolescents (male = 482, female = 406) participated in this survey. Their mean age was 15.9 years. Attrition was 93.7% in the first year (832 respondents; Mage = 15.91, SDage = 0.41; male = 54.0%, female = 46.0%). Attrition was 91.1% in the second year (809 respondents; Mage = 16.90, SDage = 0.40; male = 53.4%, female = 46.6%). In the third year, attrition was 88.4% (785 respondents; Mage = 17.90, SDage = 0.40; male = 53.1%, female = 46.9%).

### 3.3. Instrument 

Career maturity. The 21-item measure with a 6-point scale (not at all = 1 to very much = 6) developed by KEIS was used [15]. We conducted an exploratory factor analysis to identify the factor structure. Based on the Kaiser–Meyer–Olkin value (0.86) and Bartlett’s test (*p* < 0.001), the data were judged appropriate for factor analysis. The results of applying maximum likelihood estimation and direct oblique rotation indicated that a four-factor structure was appropriate—the first factor (career preparation) accounted for 27.19% of the total variance, a second factor (career plan) explained for 10.06%, a third factor (career capacity) explained for 9.64%, and the fourth factor (career self-determination) explained 5.22%. As a result of the reliability analysis, two items (“It is better to follow the decisions of adults when choosing a career”; “I am worried because the job I want is a job that requires a lot of study”) were removed because their reliability was below 0.20; thus, 19 items were used for analysis. A higher total score indicated a higher level of career maturity. Examples of the items are as follows: “I have a plan to have the job (major) I want”; “When I make career choices, my thoughts are more important than the opinions of others”; “I have never thought about my career”; “I have a clear career plan compared to my peers”; “I am thinking about what to do now to achieve my future dreams”; “It is too early to think about my career now”. The Cronbach’s alpha for each year was 0.83, 0.83, and 0.84.

Academic Achievement. Academic performance in high school was measured in six items (Korean language, math, science, society, foreign language, and others) using a 5-point scale (upper, mid-upper, middle, mid-low, low). Upper level refers to grades within the 10th percentile, mid-upper level grades range from the 11th to 30th percentile, the middle level ranges from the 31st to 70th percentile, the mid-low level is from the 71st to 90th percentile, and lower level grades are within the 91st to 100th percentile. In this study, the mean value divided by 6 was used for the analysis. A grade point average (GPA) was used to measure academic achievement in college. The GPA was measured on a 5-point scale: A− to A+ = 5, B− to B+ = 4, C− to C+ = 3, D− to D+ = 2, F = 1.

### 3.4. Analysis

This study applied autoregressive and cross-lagged (ARCL) modeling to identify the bidirectional effects between academic achievement and career maturity by examining autoregressive and cross-lagged effects. An autoregressive effect indicates the stability of a construct over time; for example, how much variance of career maturity at time 2 is explained by career maturity at time 1? A cross-lagged effect indicates the long-term prediction of a construct at time 1 on another construct at time 2 while adjusting for the autoregressive component. In particular, a researcher can verify whether a cross-lagged effect is indicated in both directions (e.g., whether academic achievement at time 1 predicts career maturity at time 2 while career maturity at time 1 predicts academic achievement at time 2) or only one direction. In addition, ARCL could be used to test the relative strength of the cross-lagged effect (e.g., is the effect of career maturity at time 1 on academic achievement at time 2 stronger than that of academic achievement at time 1 on career maturity at time 2?).

In order to analyze autoregressive and cross-lagged effects, the researcher should verify three homogeneity hypotheses. First, in order to test the homogeneity of measurement, the model-constrained factor coefficients of the same items for the same latent variables at each time point are compared with the model without constraints (basic model). Second, the researcher should verify the homogeneity of the path such that the regression coefficients between latent variables are the same over time. Third, by using the constrained covariance between the errors set at each time point, it is possible to examine whether the relevance between latent variables over time occurred by chance. The eight models assumed in this study are as follows:

Model 1: Basic model (no-constraint model); Model 2: Homogeneity constraint on the measurement coefficient of academic achievement; Model 3: Homogeneity constraint on the measurement coefficient of career maturity; Model 4: Homogeneity constraint on the autoregressive coefficient of academic achievement; Model 5: Homogeneity constraint on the autoregressive coefficient of career maturity; Model 6: Homogeneity constraint on the cross-regression coefficient of academic achievement; Model 7: Homogeneity constraint on the cross-regression coefficient of career maturity; Model 8: Homogeneity constraint on the error coefficients of academic achievement and career maturity.

**Figure 1:**
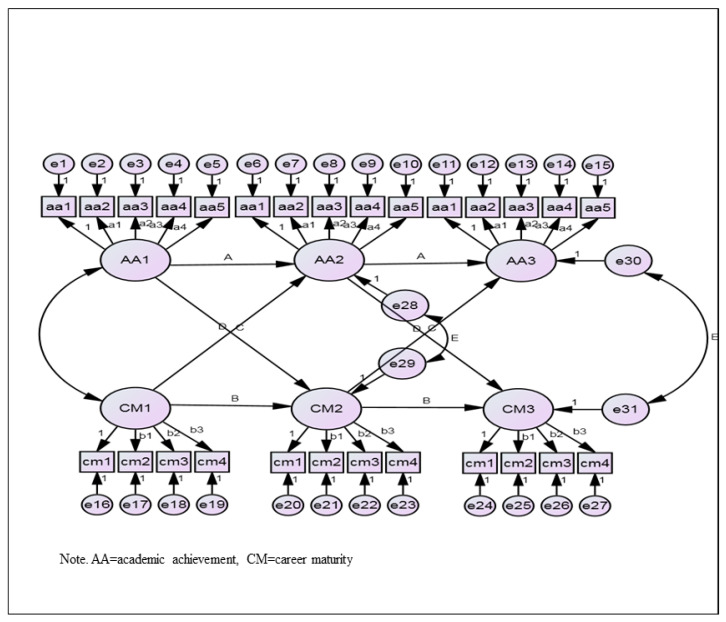
Autoregressive and cross-lagged model.

The research model is presented in Figure 1. We used the AMOS 20.0 program and chi-square, GIF, IFI, CFI, and RMSEA to evaluate the fit of the suggested model. As chi-square is sensitive to sample size, it is likely to reject the null hypothesis. Therefore, the use of other combinations of fit indices should be considered. If the GFI, IFI, and CFI are above 0.90, and the RMSEA is below 0.50, the fit of the model is acceptable.

## 4. Results

### 4.1. Descriptive Statistics

Table 1 indicates the response rate, respondents, mean age, and gender ratio at each time point. Table 2 shows the mean, standard deviation, and maximum/minimum values of the variables measured at each wave.

### 4.2. Autoregressive and Cross-Lagged Analysis

In this study, the eight models were compared sequentially, and the results are presented in Table 3. Researchers should consider the other fit indices, as χ^2^ is sensitive to the sample size in model comparisons. Cheung and Rensvold [16] proposed CFI differences as an alternative method to the χ^2^ difference test, such that if the differences in CFI values do not exceed 0.01, there is no difference between the models.

First, there was no difference between χ^2^ and the CFI value in the comparison between Models 1 and 2, which secured the homogeneity of measurement for academic achievement. Comparison between Models 2 and 3 found no difference between χ^2^ and the CFI value, which ensured the homogeneity of measurement for career maturity, and comparing Models 3 and 4 found no difference between χ^2^ and the CFI value, securing the homogeneity of autoregression for academic achievement. Comparison of Models 4 and 5 yielded a χ^2^ value of 17.534, which is larger than the critical value (3.84) for one degree of freedom. However, the CFI difference was −0.001, confirming the homogeneity of autoregression for career maturity. There was no difference when comparing Models 5 and 6, which ensured the homogeneity of cross-lagging for academic achievement, or when comparing Models 6 and 7, confirming the homogeneity of cross-lagging for career maturity. Finally, there was no difference between Models 7 and 8, which ensured the homogeneity of error covariance.

As a result of model comparison, Model 8 was selected as the final model with acceptable fit indices (χ^2^ = 462.402, df = 282, *p* < 0.001, TLI = 0.973, CFI = 0.978, RMSEA = 0.027). The results for each path coefficient are presented in Table 4. First, the autoregressive coefficients of academic achievement at a previous time point had positive effects on academic achievement at the next time point (β = 0.559, t = 21.864, *p* < 0.001). In addition, the autoregressive coefficients of career maturity at a previous time point had positive impacts on career maturity at the next time point (β = 0.527, t = 14.806, *p* < 0.001). Second, the cross-lagged effect of academic achievement on career maturity was found to be positive (β = 0.259, t = 2.201, *p* < 0.05), which indicates that higher academic achievement at a previous time point was related to higher career maturity at the next time point. In addition, the cross-lagged effect of career maturity on academic achievement was found to be positive (β = 0.023, t = 3.574, *p* < 0.001). This indicates that higher levels of career maturity at previous time points were associated with higher academic achievement at the next time points. These results indicate that there is a bidirectional relationship between academic achievement and career maturity over time. Finally, the effect of career maturity at one time point on academic achievement at the next time point was stronger than that of academic achievement at one time point on career maturity at the next time point.

## 5. Discussion

The purpose of this study was to analyze the causal relationship between academic achievement and career maturity using autoregressive cross-lagged modeling. The main results and implications of this study are as follows:

Through autoregressive analysis of the effects of academic achievement and career maturity, it was found that the autoregressive effect of academic achievement and career maturity continued stably over time. These results indicate that students with high academic achievement maintain high academic achievement across time. In addition, students with a high level of career maturity maintain a high level of career maturity across time.

A cross-lagged effect between academic achievement and career maturity was revealed. In other words, academic achievement at one time had a positive effect on career maturity at the next time point, and career maturity at one time had a positive impact on academic achievement at the next time point. These results indicate that higher academic achievement leads to higher levels of career maturity, just as a higher level of career maturity causes higher academic achievement.

The results of this study support the results of previous studies [2,3] that indicate career maturity positively affects academic achievement. Students who have a better understanding of their career options and have specific career-related plans are active in seeking out the goals and opportunities associated with their desired career paths through their studies and, consequently, are more likely to have better academic performance [6].

Many past studies argued that academic achievement is a key predictor of career maturity [5]. Academic achievement is closely related to career choice, and high academic achievement enables career choice in a variety of areas (e.g., law school, medical school). Students who have high academic achievement tend to have high self-efficacy and actively explore their careers. Academic achievement may stimulate career exploration, and consequently, it can help students to make the best career choice through consideration of a match between their aptitudes and careers. In particular, academic achievement in the education-oriented society of Korea may have a great influence on students’ career development, and the results of this study may reflect a specific feature of Korean society.

Many studies have verified that academic achievement has a positive impact on career maturity, while other researchers have argued that career maturity is a key predictor of academic achievement. The contribution of this study was to verify the long-term reciprocal association between career maturity and academic achievement. In addition, the results indicate that the effect of career maturity on academic achievement is stronger than that of academic achievement on career maturity. Chung, Lee, and Ahn [2] examined the longitudinal relationship between career maturity, academic engagement, and academic achievement in elementary, middle, and high school students. By using a multivariate latent growth modeling, it was found that career maturity had a positive effect on academic achievement by mediating academic engagement. The results hold implications for career guidance.

These results suggest that parents, teachers, and psychologists need to apply the concept of career maturity to the career guidance of adolescents. Parents and teachers should be more interested in students’ interests and aptitudes as well as academic achievement and help them explore the information needed to make a decision regarding their careers, and help them choose a career by themselves, rather than over-emphasizing only academic performance and college entrance. It is important to recognize that these efforts will lead students to choose a college major in accordance with their aptitudes, and in turn, improve academic achievement in college and increase life satisfaction [17]. In addition, career maturity and career development are influenced by parents’ perspectives on occupation [6]. Therefore, parents should talk frequently with their children about careers and the value of various jobs.

One of the effective ways to increase the level of career maturity among students is through career counseling. Schools in many countries are supporting career counseling through professional counseling systems [5,18]. Career counseling may help students effectively explore their career options and specifically prepare for their careers [19]; it may increase the self-efficacy of career choice by helping students realize the importance of internal standards, such as aptitude and interest, in choosing a career. Understanding the importance of internal standards for career choice is one of the core concepts of career maturity [20]. Therefore, a career counseling service needs to be further expanded in middle schools, high schools, and colleges, along with the development of more effective career counseling programs. The results suggest that career development, and career maturity, need to be included in formal and informal curricula in middle school, high school, and college, to improve the academic achievement of students.

This study analyzed the bidirectional relationship between career maturity and academic achievement among other issues, focusing on adolescents. Academic achievement and career maturity may be particularly important issues for Korean youth. For Korean adolescents, academic achievement is highly emphasized, but career exploration and career maturity are less important. The results of this study suggest that career maturity can be closely related to academic achievement, and career maturity for Korean adolescents may play a decisive role in their career development.

This study was based on the data of high school students, so one of the limitations is the generalizability of the current research results to early adults such as college students. In addition, academic achievement was measured by self-report, which requires the use of official academic records. In future studies, it is necessary to try to expand the model by including other variables (e.g., job satisfaction). In addition, because career counseling may have a significant impact on career maturity, future studies need to verify the relationship between career counseling, career maturity, and academic achievement.

## 6. Conclusions

This study used a longitudinal analysis with an autoregressive cross-lagged model to examine the causal relationship between academic achievement and career maturity. The result showed that there was a bidirectional influence between academic achievement and career maturity. The results indicate that career maturity has a positive effect on academic achievement, and academic achievement can cause career maturity. The results of this study imply that career awareness and exploration through career education in the educational field can have a positive influence on adolescents’ academic achievement, and it is necessary to include career exploration and planning in the informal curriculum. Of course, these topics are not the goals of compulsory education, and good academic achievement and good career maturity may not be decisive in the development of vocations. Nevertheless, considering the characteristics of Korean society—namely, it overemphasizes academic achievement and relatively deals less with career exploration—the results of this study have great implications for education policy in Korean society. Based on past studies that academic achievement and career maturity can act as protective factors for mental health problems such as depression and anxiety, the results of this study imply that improvement in career maturity may contribute to the maintenance of mental health among adolescents.

## Figures and Tables

**Table 1:** Response rate, respondents, mean age, and gender ratio N = 888.

Year	Response Rate	Respondents	Age		Gender	
			Mean	SD	Male	Female
2008	93.7%	832	15.91	0.41	54.0%	46.0%
2009	91.1%	809	16.90	0.40	53.4%	46.6%
2010	88.4%	785	17.90	0.40	53.1%	46.9%

**Table 2:** Mean and standard deviation of variables.

	Min	Max	Mean	SD	N
Academic achievement 1	1	5	3.27	0.92	888
Academic achievement 2	1	5	3.28	0.81	888
Academic achievement 3	1	5	3.29	0.78	888
Career maturity 1	48	112	78.59	10.87	888
Career maturity 2	26	112	80.24	10.54	888
Career maturity 3	51	113	81.67	10.53	888

**Table 3:** The fitness of research model.

Model	χ^2^ (df)	df	TLI	CFI	RMSEA
Basic model	423.698	263	0.974	0.981	0.026
Measurement homogeneity (academic achievement)	436.610	271	0.974	0.980	0.026
Measurement homogeneity (career maturity)	440.214	277	0.975	0.980	0.026
Autoregressive homogeneity (academic achievement)	440.879	278	0.975	0.980	0.0.026
Autoregressive homogeneity (career maturity)	458.413	279	0.973	0.979	0.027
Cross-lagged homogeneity (academic achievement)	458.906	280	0.973	0.979	0.027
Cross-lagged homogeneity (career maturity)	461.946	281	0.973	0.978	0.027
Error variance homogeneity	462.402	282	0.973	0.978	0.027

**Table 4:** Path coefficients of cross-lagged model.

Path	Unstandard Coefficient	Standard Coefficient	Standard Error	t
AA 1 wave → AA 2 wave	0.559	0.550	0.026	21.864 ***
AA 2 wave → AA 3 wave	0.559	0.594	0.026	21.864 ***
CM 1 wave → CM 2 wave	0.527	0.613	0.036	14.806 ***
CM 2 wave → CM3 wave	0.527	0.449	0.036	14.806 ***
AA 1 wave → CM 2 wave	0.259	0.066	0.118	2.201 *
AA 2 wave → CM 3 wave	0.259	0.066	0.118	2.201 *
CM 1 wave → AA 2 wave	0.023	0.103	0.006	3.574 ***
CM 2 wave → AA 3 wave	0.023	0.094	0.006	3.574 ***

* *p* < 0.05, *** *p* < 0.001, CM = career maturity, AA = academic achievement.

## Data Availability

Youth Panel Survey data of the Korea Employment Information Service are available at http://hdl.handle.net/20.500.12236/13795, accessed on 22 March 2020.
